# Retinal Microvasculature Image Analysis Using Optical Coherence Tomography Angiography in Patients with Post-COVID-19 Syndrome

**DOI:** 10.3390/jimaging9110234

**Published:** 2023-10-24

**Authors:** Maha Noor, Orlaith McGrath, Ines Drira, Tariq Aslam

**Affiliations:** 1 Department of Eye Research, Manchester Royal Eye Hospital, Manchester University NHS Foundation Trust, Oxford Road, Manchester M13 9WL, UK; 2 Ophtalmologie Département, Centre Hospitalier Universitaire de Toulouse, 31300 Toulouse, France; 3 Division of Pharmacy and Optometry, School of Health Sciences, Faculty of Biology, Medicine and Health, The University of Manchester, Oxford Road, Manchester M13 9PL, UK

**Keywords:** post-COVID-19 syndrome (PCS), long COVID, optical coherence tomography angiography (OCT-A), SD-OCT, nerve fibre layer, ganglion cell layer, retina

## Abstract

Several optical coherence tomography angiography (OCT-A) studies have demonstrated retinal microvascular changes in patients post-SARS-CoV-2 infection, reflecting retinal-systemic microvasculature homology. Post-COVID-19 syndrome (PCS) entails persistent symptoms following SARS-CoV-2 infection. In this study, we investigated the retinal microvasculature in PCS patients using OCT-angiography and analysed the macular retinal nerve fibre layer (RNFL) and ganglion cell layer (GCL) thickness via spectral domain-OCT (SD-OCT). Conducted at the Manchester Royal Eye Hospital, UK, this cross-sectional study compared 40 PCS participants with 40 healthy controls, who underwent ophthalmic assessments, SD-OCT, and OCT-A imaging. OCT-A images from the superficial capillary plexus (SCP) were analysed using an in-house specialised software, OCT-A vascular image analysis (OCTAVIA), measuring the mean large vessel and capillary intensity, vessel density, ischaemia areas, and foveal avascular zone (FAZ) area and circularity. RNFL and GCL thickness was measured using the OCT machine’s software. Retinal evaluations occurred at an average of 15.2 ± 6.9 months post SARS-CoV-2 infection in PCS participants. Our findings revealed no significant differences between the PCS and control groups in the OCT-A parameters or RNFL and GCL thicknesses, indicating that no long-term damage ensued in the vascular bed or retinal layers within our cohort, providing a degree of reassurance for PCS patients.

## 1. Introduction

In March of 2020, the severe acute respiratory syndrome coronavirus 2 (SARS-CoV-2) gave rise to a global pandemic, incurring detrimental effects on the health, economy, and social infrastructures of populations worldwide. The effects of the SARS-CoV-2 infection, termed acute coronavirus disease 2019 (COVID-19), range from pneumonia and acute respiratory distress syndrome (ARDS) to thromboembolic disease, septic shock, and multi-organ failure [1]. In the aftermath of the pandemic, a post-viral sequela of SARS-CoV-2 infection emerged, referred to as post-COVID-19 syndrome (PCS) [2] or post-COVID-19 condition [3].

Post COVID-19 syndrome refers to the persistence of certain clinical symptoms more than 12 weeks after the initial COVID-19 infection, which cannot be explained by an alternative diagnosis [4]. Persistence of symptoms 4 or more weeks after the infection may be referred to as post-acute sequelae of SARS-CoV-2 infection (PASC) [5] or ‘Long COVID’. Symptoms of PCS may include but are not limited to fatigue, dyspnoea, cough, autonomic symptoms (chest pain, palpitations, tachycardia), neurocognitive impairment i.e., ‘brain fog’, arthralgia, myalgia, headaches, anosmia, ageusia, gastrointestinal disturbances, sleep disturbances, hair loss, and psychiatric disorders such as depression and anxiety [5]. Immune dysregulation, autoimmunity, dysautonomia, viral persistence, re-activation of latent viral pathogens, neutrophil extracellular traps (NETs), dysregulation of the renin-angiotensin-aldosterone system (RAAS), coagulopathies (hypercoagulation, thrombosis), fibrin amyloid micro-clots, endothelial dysfunction, and impaired microvasculature, are some of the principle pathophysiological mechanisms underpinning post-COVID-19 syndrome described in the literature to date [5,6,7,8,9,10,11,12,13,14,15,16,17,18,19,20,21,22,23,24,25,26,27,28,29,30,31,32,33,34]. 

Studies have shown that increasing age, female sex, lower socioeconomic status, obesity, presence of co-morbidities, and smoking are associated with an increased risk of development of PCS [35,36,37,38,39,40]. It has been estimated that, globally, approximately 15% of COVID-19 patients experience persistent symptoms at 12 months following acute SARS-CoV-2 infection [41]. In the UK, 3.1% of the population self-reported suffering with long-term symptoms following their initial COVID-19 infection between September 2022 and January 2023 [42]. The worldwide influence of COVID-19 affects not only personal mental and physical well-being, but also social, economic, and productivity-related aspects in the healthcare, finance, and employment sectors. Decreased productivity, the requirement for medical assistance, and the growing need for diagnosis render post COVID-19 syndrome a deserving candidate for investment in the healthcare industry [43]. Therefore, it is important to investigate and research all characteristics of this condition.

SARS-CoV-2 enters cells via the angiotensin-converting enzyme-related carboxypeptidase (ACE2) receptor [44], which is also expressed on the surface of neuroretinal cells including Muller cells, retinal pigment epithelium, and pericytes of retinal and choroidal endothelial cells [45]. Whilst the most common ophthalmic manifestations of acute SARS-CoV-2 infection consist of conditions such as conjunctivitis and anterior uveitis [46,47,48], scleritis, episcleritis, inflammatory orbital diseases (dacryoadenitis, orbital cellulitis, mucormycosis), optic neuritis, papillophlebitis, cranial nerve palsies, choroiditis, retinitis, retinal vasculitis [48,49,50,51], and retinal artery and vein occlusion have also been reported [52]. Retinal examination in patients following COVID-19 infection has demonstrated a wide array of clinical signs ranging from cotton wool spots (CWS), retinal micro- and macro-haemorrhages, and venous tortuosity [51,53,54] indicative of acute vascular events and retinal ischaemia. One of the first studies pertaining to optical coherence tomography (OCT) imaging of the retina in patients with COVID-19 identified hyper-reflective plaques in the ganglion-cell-inner plexiform layer (GC-IPL) [54]. However, subsequent publications attributed this finding to normal variations in the retinal vasculature [55,56]. Increased peri-papillary retinal nerve fibre layer (RNFL) thickness [57,58], increased central macular thickness, and decreased ganglion cell layer and inner nuclear layer thickness have also been reported in patients following COVID-19 infection [59]. Therefore, SARS-CoV-2 infection can induce microstructural changes in the retina, which may persist long-term in patients with post-COVID-19 syndrome. Given that the retina and optic disc are considered to be intraorbital extensions of the central nervous system [60], alterations may prevail especially in those with neurocognitive symptoms.

The eye can be considered as a window into the body’s microvascular system. Optical coherence tomography angiography (OCT-A) offers a non-invasive opportunity to analyse the retinal circulation in vivo, providing insight into the subject’s systemic microvasculature by inference [61]. Several studies have investigated the retinal vasculature of patients infected with COVID-19 using OCT-A to date. A key finding of note is reduction in the macular vessel density from as early as 2 weeks following the infection up to 8 months afterward [62,63,64,65,66,67,68,69,70,71,72,73,74,75]. 

Considering the overwhelming amount of literature reporting an alteration in the retinal microvasculature in patients with a history of COVID-19 illness, it is pertinent to investigate whether these effects last long term, especially in patients with ongoing symptoms of post-COVID-19-syndrome. Furthermore, there is currently a paucity of literature examining the retinal vasculature of patients with PCS. Therefore, the primary aim of this study is to investigate the retinal microvasculature of patients with post-COVID-19 syndrome using OCT-angiography, in order to determine the long-term sequelae of SARS-CoV-2 infection on retinal tissues. Additionally, using spectral domain-OCT (SD-OCT) the thickness of the macular retinal nerve fibre layer and macular ganglion cell layer will also be examined, to determine if any anatomical alterations coincide with ongoing symptoms of PCS, particularly neurocognitive symptoms. 

## 2. Materials and Methods

This was a prospective, cross-sectional observational study, conducted at the Manchester Royal Eye Hospital (MREH), UK. Ethical approval was obtained from the Health Research Authority (HRA) and Health and Care Research Wales (HCRW) along with the Office for Research Ethics Committees Northern Ireland (ORECNI). The study was conducted with adherence to the Declaration of Helsinki; written informed consent was obtained from all participants. 

### 2.1. Participant Recruitment

Patients were recruited into two distinct groups for this comparative study. The first group comprised of patients over 18 years of age with an established clinical diagnosis of post-COVID-19 syndrome by the respiratory team at Manchester Royal Infirmary, UK. All included subjects either had a reverse transcription-polymerase chain reaction (RT PCR)—confirmed diagnosis of COVID-19 at an earlier stage or a clinical diagnosis of COVID-19 (as testing was not readily available in the early stages of the pandemic in the UK). Patients recruited within the post-COVID-19 syndrome cohort may have an initial mild, moderate, or severe initial illness with or without requirement for hospitalisation or outpatient treatment, allowing us to examine the retinal microvasculature in a wider range of PCS participants. 

The second group, the controls, included patients over 18 years of age who did not have a recent history of COVID-19 infection or a diagnosis of post-COVID-19 syndrome. For both groups, we excluded patients with a history of diabetes, uncontrolled hypertension, stroke, haematological disorders, neurodegenerative diseases, high myopia or hypermetropia (above ± 6 dioptres), high astigmatism (>3 dioptres), significant media opacity compromising fundus imaging, or signs or previous history of choroidal atrophy, exudative age-related macular degeneration (AMD), central serous chorioretinopathy, glaucoma, acquired and hereditary optic neuropathy, hereditary retinal diseases, demyelinating disorders, and keratoconus. Both cohorts were age and sex matched. Recruitment commenced in April 2021 and extended to March 2023 due to delays caused by the COVID-19 pandemic. 

Clinical history taking, visual acuity measurement, and OCT-A imaging was undertaken for each participant of the study. The clinical history taking comprised of details of the participants’ acute COVID-19 illness, method of acute COVID-19 diagnosis (i.e., clinical or by PCR testing), disease course, ongoing symptoms of post-COVID-19 syndrome, vaccination history, relevant past medical history, smoking history, Body Mass Index (BMI) and HbA1c (if available), past ocular history, and ophthalmic prescription. The best corrected visual acuity was measured using Early Treatment Diabetic Retinopathy Study (ETDRS) charts at four metres, converted to the Logarithm of the Minimum Angle of Resolution (LogMAR). 

### 2.2. OCT Imaging

Each patient then underwent wide-field (10 × 10 mm) macula and foveal (4 × 4 mm) OCT-A imaging using the Canon Xephilio OCTA-1 machine (Canon Medical Systems Europe B.V©, Amstelveen, The Netherlands). The field of investigation was centred on the foveal region. Scans had a 10-layer automated segmentation and a refresh rate of 70,000 A-scans/s. The depth of field of view was set to 10 × 10 mm and 4 × 4 mm with an axial sampling density of 464 × 464 px, with the number of repetitions set at two. For the purposes of this study, only the retinal superficial capillary plexus (SPC), which provided the most consistently high-quality images, was examined. Both eyes were imaged in each participant, however only the highest quality eye image was used for analysis. Inter-eye correlations and statistical complexities highlighted by Murdoch et al. would allow for us to include both eyes into the study, albeit with more complex and less easily recognised and interpreted techniques [76]. In this study however, image quality for accurate OCT-A measures, as noted by Czako et al., was of paramount importance [77]. Notably, we were aware that many PCS participants suffered with dry eyes, fatigue, and dyspnoea, and we opted to include the highest quality imaged eye only for each patient in this study protocol with standard statistical techniques to optimise overall image analysis validity. Lubricants were offered to all patients to mitigate effects of any dry eye disease. Pharmacological mydriasis was attained (tropicamide 1%) in cases where the quality of imaging was affected by lack of pupillary dilatation. The image acquisition technique was regimented, in that all patients were instructed to focus on the cross shape in the OCT-A machine to ensure standardisation of the macular image procured. Stability of the head was ensured, and all images were captured in dim lighting.

A two-fold strategy was employed to evaluate the quality of the images obtained. Initially, the Canon Quality Index from the OCT-A machine was utilised, accepting only images with an index of ≥7. Additionally, imagers with clinical expertise and a senior consultant conducted real-time evaluations of the OCT-A and OCT images to identify significant segmentation errors, and shadow or motion artefacts, leading to image exclusion if detected. The OCT-A images obtained from both participant groups (one eye per patient) were then analysed using an in-house specially designed image-processing software, OCT-A Vascular Image Analysis (OCTAVIA), which carried out an automated analysis and uploaded the specified measurements to a central Microsoft^®^ Excel^®^ 2021 spreadsheet. 

**Figure 1:**
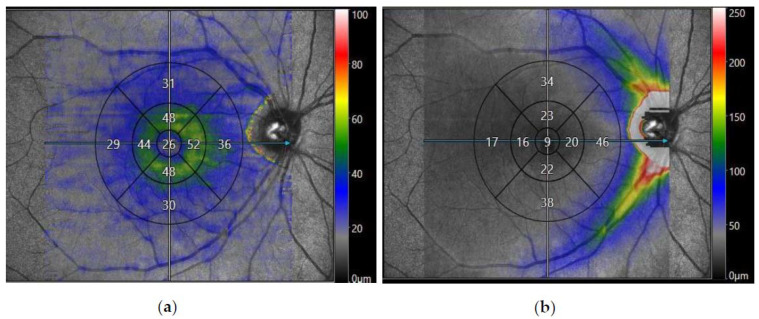
Spectral domain-optical coherence tomography (SD-OCT) of the macula obtained from Canon Xephilio OCT-A1 Machine (Canon Medical Systems Europe B.V©, Amstelveen, Netherlands) displaying a 10 × 10 mm macular image from a participant with post-COVID-19 syndrome segmented into nine EDTRS zones. The segments consist of superior outer, superior inner, nasal outer, nasal inner, inferior outer, inferior inner, temporal outer, temporal inner, and foveal (central) zones. (**a**) Displays the average thickness of the macular retinal nerve fibre layer (mRNFL) in nine EDTRS zones. (**b**) Displays the average thickness of the macular ganglion cell layer (mGCL) in nine EDTRS zones.

Spectral domain OCT of the macula was also performed to analyse the average thicknesses of the macular retinal nerve fibre layer (mRNFL) and macular ganglion cell layer (mGCL) in microns. The OCT machine’s internal software segmented the 10 × 10 macular image into the nine zones specified by the Early Treatment Diabetic Retinopathy Study (ETDRS) [78], giving an average value of RNFL or GCL thickness in each zone, shown in Figure 1. These values were exported into a Microsoft^®^ Excel^®^ 2021 spreadsheet, and a mean value for the thickness of the outer and inner segments was calculated. The final study parameters included the mean thickness of the outer segment, inner segment, and foveal (central) region of the mRNFL and mGCL. 

### 2.3. OCT-Angiography Image Processing Algorithm

The OCTAVIA algorithm was programmed using MATLAB^®^ 2021 by the corresponding author (TA), developed from previously published work on small field OCT-A imaging in diabetic retinopathy (DR) [79]. Additional evidence for its reliability and validity are provided in Appendix A, Appendix A.1 Reliability and validity of the software.

OCT-angiography has demonstrated a range of retinal vascular changes, including enlargement of the foveal avascular zone (FAZ) and reduced macular vessel density, in diabetic retinopathy [80,81] but also specifically in patients with recent SARS-CoV-2 infection [62,63,64,65,66,67,68,69,70,71,72,73,74,75].For our study we chose outcome measures to reflect a comprehensive but relevant assessment based upon previous research and clinical experience. The final measured parameters are listed in Table 1. 

Large vessel and capillary intensity refer to the amount of blood flow through the large and capillary vessels, respectively. The percentage capillary network or vessel density is an index of vascularity, indicating the retinal area occupied by vessels divided by the total retinal area. Foveal avascular zone (FAZ) refers to the foveola and immediate parafoveal retina which lacks capillaries, relying on blood supply from the choriocapillaris. In addition to enlargement of the area of the FAZ, distortion of its circularity has also been observed in DR [82], leading us to examine this parameter to investigate the retinal microvasculature in detail. 

**Figure 2:**
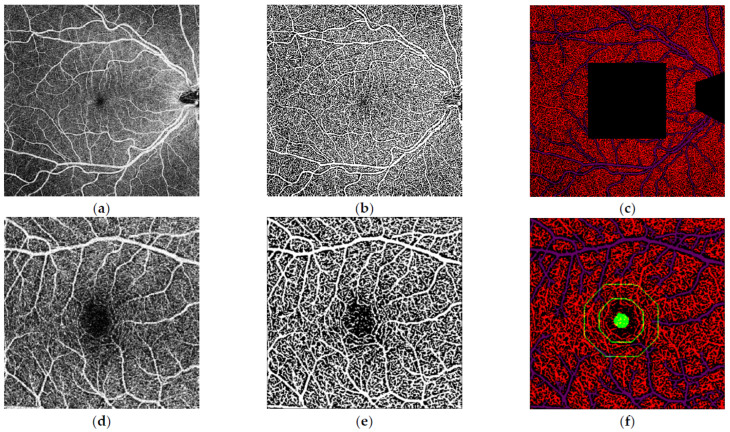
Analysis of the macular 10 × 10 mm and 4 × 4 mm optical coherence tomography-angiography (OCT-A) images performed by our inhouse software. (**a**) 10 × 10 mm macular OCT-Angiography image of the right eye. (**b**) Binarisation of the 10 × 10 mm macular OCT-A image as a processing step. (**c**) Final segmentation of the image following removal of optic disc and the central 4 × 4 mm area which was analysed in separate dedicated 4 × 4 mm images (**d**) 4 × 4 mm macular OCT-Angiography image of the right eye. (**e**) Binarisation of the 4 × 4 mm macular OCT-A image. (**f**) Final segmentation of the 4 × 4 mm image with parafoveal and perifoveal zones highlighted.

To perform image analysis, OCTAVIA has two distinct processes, using both 4 × 4 and 10 × 10 images, shown in Figure 2. The software firstly inputs the subject’s 10 × 10 mm macular image, followed by the OCT-A machine’s own in-built proprietary binary interpretation of the same image. This process is repeated for the 4 × 4 mm foveal image. The 4 × 4 mm image allows for greater detail of the foveal avascular zone (FAZ) aiding more accurate measurement of its parameters. The proprietary binary images provide a template for accurately deriving the SCP vasculature. Morphological processing techniques are utilised to distinguish between larger and smaller vessels and thresholds applied onto processed images to identify low-intensity i.e., ischaemic areas. The large and capillary vessel intensity is measured by measuring the intensity of the pixels within the skeletonised vessels. During the initial software development phase, it became apparent that small vitreous opacities can cause darkened patches on the OCT-A images. To prevent the software misinterpreting areas of such low signal, including the optic disc, as ischaemia, the functionality to manually crop out these observed areas and the optic disc was integrated into an initial pre-processing step. The final data are directly outputted to a central Microsoft^®^ Excel^®^ spreadsheet by the software.

### 2.4. Statistical Methodology

The sample size for this study was calculated based on data collected in a previous study on diabetic retinopathy and OCT-angiography [79] and is provided in full in Appendix A, Appendix A.2 Sample size calculation. We opted to calculate the sample size using data from a cohort of patients with diabetic retinopathy because the alterations observed in OCT-A studies in patients with diabetic retinopathy are comparable to those seen in the early stages of COVID-19 infection, such as the enlargement of the FAZ [80]. A minimum of 31 participants in each group were calculated. Therefore, we aimed to have 40 participants in each of our study cohorts (PCS and controls). 

Statistical analyses were performed using jamovi (version 2.3) [83]. Normality was assessed using the Shapiro–Wilk test, histograms, and Q-Q plots. Quantitative variables following a normal distribution were studied with Student *t*-tests, while those without a normal distribution were analysed using the Mann–Whitney U-test. The adjusted *p* value was set at <0.00357 after Bonferroni correction for 14 study parameters. A linear regression was also undertaken, evaluating the effect of dependent variables including age, gender, and length of time since initial COVID-19 infection on a key outcome variable mean capillary intensity (10 × 10 mm OCT-A image). 

## 3. Results

### 3.1. Demographic Distribution

A total of 80 eyes of 80 patients were included in this study (44 right eyes and 36 left eyes). There were 40 patients included in the group with post-COVID-19 syndrome and 40 controls. The PCS group was comprised of 31 females and 9 males, with an average age of 47.8 ± 10.4 years. Ethnic distribution consisted of thirty-five Caucasians, two Asians, one Black Caribbean and one Mixed Caucasian and Black Caribbean participant. Clinical assessment including clinical history, visual acuity measurement, OCT and OCT-A imaging was conducted at an average of 15.2 ± 6.9 months (range 3–32 months) after the initial SARS-CoV-2 infection. The control group was comprised of twenty-seven females and thirteen males, with an average age of 44.0 ± 14.6 years. Ethnic distribution consisted of thirty-four Caucasians, five Asians, and one Black African participant. No significant differences in age (*p* = 0.107) or sex (*p* = 0.317) distribution were found between the two groups. The mean LogMAR visual acuity was −0.0045 ± 0.168 in the PCS cohort, and 0.01652 ± 0.137 in the control subjects (*p* = 0.302). The average quality of the OCT-angiography 10 × 10 mm images was 7.40 ± 0.67 in the PCS cohort and 7.55 ± 0.64 in the controls (*p* = 0.465). Average quality of 4 × 4 mm images was 7.68 ± 0.76 in the PCS group and 7.83 ± 0.90 in the controls (*p* = 0.465). The quality of the SD-OCT macula images was 8.18 ± 0.82 in the PCS group and 8.24 ± 0.65 in the controls (0.779). Further details can be found in Table 2. 

### 3.2. Clinical History

Participants in the PCS cohort with notable past medical histories included six obese participants, eight asthmatic individuals, six with obstructive sleep apnoea, and one with fibromyalgia. Neither the controls nor any of the PCS cohort participants had a history of diabetes or hypertension. Only one patient in the cohort had a history of prior hospitalisation for COVID-19 pneumonia, with the remainder having had a mild COVID-19 infection which did not require any inpatient or outpatient treatment. 

The most prevalent symptoms of post-COVID-19 syndrome identified during clinical history taking were fatigue (30/40), dyspnoea (23/40) of which 4/23 reported exertional dyspnoea, cognitive dysfunction termed “brain fog” (16/40), and palpitations (15/40). Four patients had been diagnosed with paroxysmal orthostatic tachycardia syndrome (POTS). Interestingly, 3/40 patients reported intermittent visual disturbance and 11/40 expressed presence of dry eyes. Table 3 provides further details on the PCS cohort’s clinical symptoms, categorised by physiological systems, adapted from a comprehensive review of PCS by NICE guidelines [84].

### 3.3. OCT-Angiography Image Analysis 

All 80 eyes were processed successfully through the OCTAVIA image analysis system, which provided output metrics as planned. No clinically observable significant macro- or micro-vascular abnormalities were detected in either participant groups by a retinal specialist (TA). The results indicated that there were no statistically significant differences between the PCS and control cohort in any of the SCP measures in the 10 × 10 images in terms of the mean large vessel intensity (*p* = 0.588), mean capillary intensity (*p* = 0.099), mean vessel densities (*p* = 0.103) and the total area of ischaemia (*p* = 0.541). In the 4 × 4 images also, no statistically significant differences were noted between the PCS and control cohort in relation to the mean vessel densities (*p* = 0.895), area of the FAZ (*p* = 0.399), and circularity of the FAZ (*p* = 0.319). 

During the study, a required remote OCT-A software upgrade occurred. Although there was no visible effect on clinical examination of the image, we explored impact of this on images and there appeared to be a possible subtle change in values of the mean capillary intensities on 4 × 4 images but of no other parameters. Therefore, for the purposes of inter-cohort and intra-cohort analysis of the mean capillary intensity (4 × 4 mm images) only individuals recruited from December 2021 (PCS cohort *n* = 20, control cohort *n* = 26) have been included. The mean capillary intensity was measured as 139.25 ± 4.32 in the PCS cohort, and 140.51 ± 6.03 in the controls (*p* = 0.350). Further details are illustrated in Table 4. 

### 3.4. OCT Analysis

#### 3.4.1. Macular RNFL and GCL Thickness 

An analysis of SD-OCT-macula images was performed in 39 participants with PCS and 34 controls following exclusion of images of inferior quality. Although increased thickness of the mean outer, inner, and foveal segments of the mRNFL was noted in the PCS cohort compared to the controls, the differences were not statistically significant. Furthermore, a reduction in the thickness of the outer and inner segment of mGCL was observed in the PCS cohort compared to the control cohort, however these findings were not statistically significant. Details provided in Table 5.

#### 3.4.2. Neurocognitive Symptoms and Macular RNFL and GCL Thickness 

Within the PCS cohort, we evaluated the thickness of the macular RNFL and GCL segments in patients with ongoing neurocognitive symptoms, encompassing cognitive dysfunction i.e., brain fog and headaches (*n* = 24) compared with PCS participants without these symptoms (*n* = 15). No statistically significant differences were noted in the thickness of the macular RNFL or GCL segments within these sub-groups, described in Table 6. 

### 3.5. Linear Regression

Linear regression analysis was undertaken to evaluate the effect of age, gender, and length of time since COVID-19 infection on mean capillary intensity (10 × 10 OCT-A image), a key measure of retinal microvasculature examined in our study. No significant relationships were observed with respect to the above independent variables on the mean capillary intensity (age, *p* = 0.922; gender, *p* = 0.966; length since initial infection, *p* = 0.332). This has been demonstrated below in Table 7. 

## 4. Discussion

Post-COVID-19 syndrome has been linked to a persistent impairment of the systemic microvasculature. This study explored the retinal microvasculature network as a potential window into the pathophysiology of post-COVID-19, considering the known homology of the retinal vascular bed with systemic diseases.

We used custom-designed image analysis algorithms to assess a range of features using the most modern retinal imaging techniques including OCT and narrow and wide-field OCT-A imaging, in patients with and without post-COVID-19 syndrome. Our study shows that there were no significant differences found in any of the comprehensive measures used between our populations of people with and without this syndrome. There were no defects or abnormalities detected in the OCT of retinal layers or OCT-A of retinal vasculature.

Most studies discussing OCT-A in relation to COVID-19 primarily concentrate on patients who were hospitalised and/or treated for COVID-19 during the early stages of recovery from the infection as opposed to those experiencing post-COVID-19 syndrome (PCS). As a result, any inferences about persistent changes in the retina may be limited in their generalisability. A prominent finding in these studies has been the reduction in the central vessel density (VD) in patients with COVID-19 infection as compared to control patients [62,63,64,65,66,67,68,69,70,71,72,73,74,75]. 

Further studies entailing a slightly longer length of time between initial infection and imaging comprise of a duration of 3 month [71], up to 4 months [72], 6 months [73,74], with the longest follow-up being at 8 months post-infection [75]. The key findings were of reduced VDs in the superficial [71,72,73,74,75], deep [71,72,73,75], and radial peripapillary plexi [73], GCL thinning [74], parafoveal RNFL thinning [73,74] and FAZ enlargement [72,74,75]. 

In contrast to the studies above, the patients in our study cohort had a longer length of time since initial SARS-CoV-2 infection (15.2 ± 6.9 months (range 3–32)). Our analyses did not demonstrate any significant reduction in vessel densities or intensities in the SCP nor any differences in the area of the FAZ. The results of our investigation are encouraging, therefore, as they may indicate that any alterations in the retinal vasculature of individuals with a recent COVID-19 disease may not necessarily be long-term.

Alternatively, our negative findings may be due to our particular cohort—Other studies have been performed on patients with a moderate-severe SARS-CoV-2 infection [62,63,64,65,67,70,71,73,74,75] whilst in our study almost all participants had experienced a milder form of the disease which did not require hospitalisation and/or outpatient treatment. 

A similar narrative to OCT-A research can be seen when assessing literature on structural OCT changes. Mavi et al. found statistically significant changes with higher central foveal, mean outer nuclear layer, and mean peri-papillary RNFL thickness in the post COVID-19 patients compared to normal [85]. Ugurlu et al. also examined SD-OCTs of 129 patients with COVID-19 infection 29 to 45 days following a positive PCR test, with findings of a statistically thinner macular RNFL and GCL layer in COVID-19 patients with neurological symptoms during the acute infection compared to those with non-neurological symptoms, no symptoms/pauci-symptoms, and control subject [86]. Interestingly, Taskiran-Sag et al. studied 40 patients 113 ± 62 (SD) days after recovering from acute COVID-19 infection. Within the COVID-19 recovered cohort, significantly reduced GCL thickness were found in patients with symptoms of cognitive disturbance and headaches [87]. More recently, Kanra et al. examined 34 eyes of 20 patients with neurological symptoms 4.3 ± 2.7 (range, 1–12) months following the initial COVID-29 infection. Thinning of the macular RFNL, the GCL, and inner plexiform layer (IPL) segments were noted [88]. In our study, analysis of the SD-OCT of 39 PCS and 34 control subjects did not demonstrate any differences in the mRNFL and mGCL between PCS and control cohorts. Furthermore, no distinguishable results were noted within the PCS cohort in patients with and without neurocognitive symptoms. It is therefore possible that structural changes noted in the retinal layers post-COVID-19 infection may not persist long-term. A summary of studies pertaining to OCT-A and OCT studies in COVID-19 can be found in Appendix B, Table A5 and Table A6. 

We opted to analyse OCT-A images of the superficial retinal plexus, noting the clinical importance of this region and improved quality of imaging compared to intermediate or deep plexi as well as its predominance as a focus of analysis in other publications. However, Schlick et al. recently explored the retinal microvasculature of patients with post-COVID-19 syndrome using OCT-Angiography and found significant changes in the intermediate capillary plexus (ICP), as compared to the controls [89]. Future studies may benefit from attention to improved imaging of intermediate and deeper plexi to assess if this effect is seen longer term. 

Overall, no long-term structural changes were noted in the retinal microvasculature pertaining to the SCP, and the thickness of the RNFL and GCL layers within our PCS cohort. Our PCS cohort comprised of patients with a predominantly mild initial COVID-19 illness, no underlying conditions known to affect the retinal microvasculature such as diabetes or hypertension, with an extended length of time since the initial infection. Therefore, our results should be interpreted with this in mind when comparing to other studies. Further examination with improved imaging of the intermediate and deep plexi and the choriocapillaris, as well as recruitment of patients with varying severity of initial disease, could further enhance our understanding of the long-term implications of COVID-19 on the retinal microvasculature in different patient groups. 

SARS-CoV-2 enters cells by binding to angiotensin-converting enzyme 2 (ACE2), downregulating its activity and causing a disruption in the signalling effects of Angiotensin II and its receptor (Angiotensin II type 1 receptor, AT_1_). This leads to an accumulation of Angiotensin II, resulting in vasoconstriction, inflammation, cellular differentiation and growth, endothelial dysfunction, formation of reactive oxidative species (ROS), and microvascular thrombosis [90]. ACE2 is expressed within multiple retinal tissues, including the vascular endothelium, making it susceptible to Ag II/AT1 signalling effects and the resulting activation of the caspase 1/inflammasome pathway, responsible for the release of inflammatory cytokines [91]. Additionally, both dysregulation of the renin-angiotensin-aldosterone system (RAAS) and inflammation have been elucidated in the aetiology of post-COVID-19 syndrome [5]. No protracted alterations in the retinal microvasculature and structural layer thickness were observed in our study group following COVID-19 infection. We postulate that retinal microvascular alterations noted in the acute period post-SARS-CoV-2 infection might be predominantly ascribed to pro-inflammatory mechanisms linked to Angiotensin II. During a profound COVID-19 infection, compounded by additional co-morbidities, this response may be amplified. This is supported by studies demonstrating increased severity and mortality of COVID-19 in patients with diabetes, hypertension, and cardiovascular disease [92]. Additionally, COVID-19 disease severity has been found to affect the presence of retinopathy as a higher incidence was reported in moderate to severe disease [51,91,93]. Therefore, it is possible that, in the presence of a diminished disease severity, no underlying comorbidities, and as the duration since the initial infection extends, these inflammatory changes subside, without the occurrence of long-term ischaemic damage.

Our study was limited predominantly by challenges in imaging and defining study populations. Due to the immobility of the ophthalmic imaging apparatus, participant recruitment faced limitations as individuals were required to visit our facility instead of us conducting assessments at their respective respiratory clinics. Challenges were also encountered in image acquisition of the participants, especially with PCS due to the debilitating symptoms encompassing the disease, including dyspnoea, fatigue, and dry eyes. Movement of the head up and down due to dyspnoea posed limitations in maintaining a still stature whilst imaging was undertaken. Dry eyes significantly increased the participants’ blink frequency, and despite provision of lubricants, interfered with image acquisition. Fatigue resulted in easy tiring during imaging, reducing the number of repetitions which could be utilised to capture high quality images. These challenges were further compounded by a required imaging software update that led to potential changes in specific scans which were not visible clinically but excluded them from image analysis. In order to address these expected challenges in imaging, we dedicated some time at the beginning of the study to explore different imaging techniques with the OCT-A camera and developed a protocol that was standardised yet gave the best possible results for both cohorts of patients. We incorporated software routines for example to negate the effect of artefacts including vitreous aberrations that could obscure some regions of wide field OCTA imaging. Therefore, despite imaging challenges, we are confident that the images that were ultimately accepted into analysis in our study were all of adequate quality in both groups.

We defined patients as clearly as possible as those with a clinical diagnosis of post-COVID-19 syndrome, who were all recruited from specialist clinics designed to treat these patients. However, we recognised the heterogeneity of patients within this group. In addition, due to practical restrictions, our control sample included patients who have had COVID-19 infection but did not develop post COVID-19 symptoms. Ideally, we would have benefitted from comparing against patients who had not had a previous COVID-19 infection. Furthermore, in an ideal situation we would have compared the scans of patients with post-COVID-19 syndrome to scans they had prior to their infection. Again, this was not possible for practical reasons within the confines of this study. 

This study is distinguishable within the literature due to a multitude of reasons. To our knowledge, it is the longest study examining the effects of SARS-CoV-2 on retinal tissues in patients up to 32 months following initial acute infection, particularly those who continue to suffer with symptoms of post-COVID-19 syndrome. Furthermore, our work is distinct as our in-house specially designed image analysis software, OCTAVIA, provides a comprehensive analysis of the data obtained from OCT-A imaging, with evidence for its reliability and validity. Parameters beyond the vessel densities are obtained, providing in-depth detail on the microvasculature of the retina, including vessel intensities, presence of ischaemia, and FAZ area and circularity measures. 

## 5. Conclusions

In this study, we have demonstrated that there were no statistically significant differences in the retinal microvasculature of patients with post-COVID-19 syndrome compared to healthy cohorts. Furthermore, no significant structural differences were observed in the macular retinal nerve fibre layer (RNFL) and ganglion cell layer (GCL) of the study participants. The findings of this study indicate that despite an extensive investigation in patients with post-COVID-19 syndrome, there were no long-term structural signs of damage after detailed analysis of this accessible microvasculature bed that is known to have homology with systemic vasculature. This may serve as some positive reassurance for patients experiencing ongoing symptoms of PCS.

## Figures and Tables

**Table 1:** Parameters evaluated in the analysis of optical coherence tomography-angiography (OCT-A) images.

10 × 10 mm Image	4 × 4 mm Image
Mean large vessel intensity	Mean capillary intensity
Mean capillary intensity	Percentage capillary network (vessel density)
Percentage capillary network (vessel density)	Area of the foveal avascular zone (FAZ)
Total area of ischaemia	Circularity of the foveal avascular zone (FAZ)

**Table 2:** Demographic details and analysis of participants in the post COVID-19 syndrome (PCS) and control cohorts.

	Total	PCS Group		Control Group		Statistical Test	Significance *p* < 0.00357
**No. of Patients**	80	40		40		Chi Squared test	0.317
**Female**	58	31		27			
**Male**	22	9		13			
		**Mean**	**SD**	**Mean**	**SD**		
**Age**		47.80	10.40	44.00	14.60	Mann-Whitney U test	0.107
**LogMAR Visual Acuity**		−0.0045	0.168	+0.0165	0.137	Mann-Whitney U test	0.302
**OCT-A 10 × 10 mm Quality**		7.40	0.67	7.55	0.64	Mann-Whitney U test	0.368
**OCT-A 4 × 4 mm Quality**		7.68	0.76	7.83	0.90	Mann-Whitney U test	0.465
**SD-OCT Macula Quality**		8.18	0.82	8.24	0.65	Mann-Whitney U test	0.779

Post COVID-19 syndrome, *PCS*; Standard Deviation, *SD*; Logarithm of the Minimum Angle of Resolution, *LogMAR*; Optical coherence tomography-angiography, *OCT-A*; Spectral-domain optical coherence tomography, *SD-OCT*. Note: Hₐ μ _Control_ ≠ μ _PCS_—The two-tailed alternative hypothesis for this study was that there is a significant difference in the measured parameters between the participants with post COVID-19 syndrome (PCS) and healthy control subjects.

**Table 3:** Categories of clinical symptoms of post-COVID-19 syndrome according to physiological systems.

Physiological System	Clinical Symptoms					
**Systemic**	**Fatigue**	**Dizziness**	**Fever**			
	30	5	0			
**Cardiopulmonary**	Dyspnoea	Chest Pain	Palpitations	Pericarditis		
	23	8	15	1		
**Upper Respiratory**	Blocked Nose	Cough	Sore Throat	Voice Changes	Laryngeal Disorders *	
	0	4	0	1	2	
**Gastrointestinal**	Nausea	Vomiting	Diarrhoea	Appetite Changes	Abdominal Pain	Weight Loss
	1	0	0	0	1	1
**Musculoskeletal**	Joint Pain	Muscle Pain	Worsened Mobility			
	3	3	0			
**Neurological Or Neuromuscular**	Headache	Hyposmia/ Anosmia	Hypogeusia/ Ageusia	Paraesthesia		
	10	3	3	1		
**Psychological**	Anxiety	Depression	Post-Traumatic Stress Disorder (PTSD)	Sleep Disturbances		
	1	2	0	2		
**Neurocognitive**	Cognitive Dysfunction i.e., Brain Fog (Reduced Memory And/Or Concentration)	Cognitive Impairment	Confusion			
	16	0	0			
**Ophthalmic**	Vision Disturbances	Dry Eyes				
	3	11				
**Auditory**	Reduced Hearing	Tinnitus				
	1	1				
**Other**	Hair Loss	Post-Menopausal Bleeding	Restless Legs			
	0	1	1			

* Sensation of Intermittent Laryngeal Obstruction; Coughing Hypersensitivity Related Inducible Laryngeal Obstruction.

**Table 4:** Analysis of optical coherence tomography-angiography (OCT-A) parameters in the post-COVID-19 syndrome (PCS) cohort compared with the control cohort.

					Shapiro-Wilk		Statistical Test	Statistic	df	*p* < 0.00357
	Cohort Category	N	Mean	SD	W	*p*				
**Mean Large Vessel Intensity (10 × 10 mm)**	PCS	40	225.133	2.978	0.967	0.298	Student’s *t*	0.5435	78.0	0.588
	Control	40	225.480	2.739	0.965	0.243				
**Mean Capillary Intensity (10 × 10 mm)**	PCS	40	136.504	5.687	0.887	<0.001	Mann-Whitney U	628		0.099
	Control	40	134.035	3.850	0.986	0.890				
**Percentage Capillary Network (Vessel Densities) (10 × 10 mm)**	PCS	40	45.637	1.296	0.978	0.632	Mann-Whitney U	630		0.103
	Control	40	44.934	1.827	0.827	<0.001				
**Total Area of Ischaemia (10 × 10 mm)**	PCS	40	203.400	680.847	0.342	<0 .001	Mann-Whitney U	763		0.541
	Control	40	198.000	683.638	0.331	<0.001				
**Percentage Capillary Network (Vessel Densities) (4 × 4 mm)**	PCS	40	41.192	1.240	0.986	0.888	Student’s *t*	−0.1327	78.0	0.895
	Control	40	41.147	1.746	0.950	0.073				
**Mean Capillary Intensity (4 × 4 mm)**	PCS	20	139.245	4.323	0.949	<0.359	Mann-Whitney U	217		0.350
	Control	26	140.512	6.028	0.795	<0.001				
**Area of Foveal Avascular Zone (FAZ) (4 × 4 mm)**	PCS	40	1917.725	405.880	0.573	<0.001	Mann-Whitney U	715		0.399
	Control	40	1925.050	389.784	0.589	<0.001				
**Circularity of Foveal Avascular Zone (FAZ) (4 × 4 mm)**	PCS	40	0.897	0.194	0.702	<0.001	Mann-Whitney U	699		0.319
	Control	40	0.869	0.202	0.775	<0 .001				

Post COVID-19 syndrome, *PCS*; Standard Deviation, *SD*; Shapiro Wilk test statistic, *W*; Degrees of Freedom, *df*. Note: Hₐ μ _Control_ ≠ μ _PCS_—The two-tailed alternative hypothesis for this study was that there is a significant difference in the measured parameters between the participants with post COVID-19 syndrome (PCS) and healthy control subjects.

**Table 5:** Analysis of the thickness (microns) of the macular retinal nerve fibre layer (RNFL) and ganglion cell layer (GCL) in the post COVID-19 syndrome (PCS) group (*n* = 39) and control group (*n* = 34).

				Shapiro-Wilk Test		Statistical Test	Statistic	df	*p* < 0.00357
Mean Thickness (Microns)	Cohort Category	Mean	SD	W	*p*				
**Mean Outer Segment mRNFL**	**PCS**	36.11	5.23	0.930	0.018	Mann-Whitney U	617		0.615
	**Control**	35.31	4.66	0.978	0.698				
**Mean Inner Segment mRNFL**	**PCS**	21.71	2.30	0.959	0.166	Student’s *t*	1.1007	71.0	0.275
	**Control**	21.18	1.78	0.945	0.085				
**Foveal (Central) Segment mRNFL**	**PCS**	8.67	1.90	0.913	0.005	Mann-Whitney U	654		0.920
	**Control**	8.65	2.52	0.928	0.027				
**Mean Outer Segment mGCL**	**PCS**	30.34	3.48	0.973	0.454	Mann-Whitney U	599		0.479
	**Control**	31.63	5.05	0.706	<0.001				
**Mean Inner Segment mGCL**	**PCS**	50.42	5.96	0.946	0.058	Mann-Whitney U	645		0.842
	**Control**	50.95	5.91	0.931	0.033				
**Foveal (Central) Segment mGCL**	**PCS**	19.85	5.94	0.865	<0.001	Mann-Whitney U	638		0.786
	**Control**	19.35	5.71	0.965	0.328				

Post COVID-19 syndrome, *PCS*; Macular Retinal Nerve Fibre Layer, *mRNFL*; Macular Ganglion Cell Layer, *mGCL*; Standard Deviation, *SD*; Shapiro Wilk test statistic, *W*; Degrees of Freedom, *df*. Note: Hₐ μ _Control_ ≠ μ _PCS_—The two-tailed alternative hypothesis for this study was that there is a significant difference in the measured parameters between the participants with post COVID-19 syndrome (PCS) and healthy control subjects.

**Table 6:** Analysis of the thickness (microns) of the macular retinal nerve fibre layer (RNFL) and ganglion cell layer (GCL) in patients with post COVID-19 syndrome (PCS) and neurocognitive symptoms (*n* = 24) compared to post COVID-19 syndrome patients without neurocognitive symptoms (*n* = 15).

				Shapiro–Wilk		Statistical Test	Statistic	df	*p* < 0.00357
Mean Thickness (Microns)	Neurocognitive Symptoms	Mean	SD	W	*p*				
**Mean Outer mRNFL**	Y	36.15	5.62	0.861	0.003	Mann-Whitney U	167		0.707
	N	36.05	4.74	0.939	0.367				
**Mean Inner mRNFL**	Y	21.79	2.35	0.917	0.050	Student’s *t*	0.2722	37.0	0.787
	N	21.58	2.28	0.957	0.647				
**Foveal (Central) mRNFL**	Y	8.88	1.73	0.906	0.029	Mann-Whitney U	157		0.510
	N	8.33	2.16	0.926	0.234				
**Mean Outer mGCL**	Y	30.61	3.10	0.929	0.091	Student’s *t*	0.6193	37.0	0.540
	N	29.90	4.09	0.954	0.581				
**Mean Inner mGCL**	Y	50.80	5.47	0.935	0.127	Student’s *t*	0.5056	37.0	0.616
	N	49.80	6.83	0.934	0.315				
**Foveal (Central) mGCL**	Y	20.54	5.16	0.870	0.005	Mann-Whitney U	129		0.140
	N	18.73	7.06	0.823	0.007				

Macular Retinal Nerve Fibre Layer, *mRNFL*; Macular Ganglion Cell Layer, *mGCL*; Neurocognitive symptoms present, *Y*; Neurocognitive symptoms not present, *N*; Standard Deviation, *SD*; Shapiro Wilk test statistic, *W*; Degrees of Freedom, *df*. Note: Hₐ μ _Y_ ≠ μ _N_—The two-tailed alternative hypothesis was that there is a significant difference in the measured parameters between the participants with post COVID-19 syndrome (PCS) with neurocognitive symptoms and those without neurocognitive symptoms.

**Table 7:** Linear regression analysis of independent variables (age, gender, length since initial infection) and dependent variable, mean capillary intensity, measured on 10 × 10 mm optical coherence tomography-angiography (OCT-A) Image.

Model	R	R^2^		
1	0.164	0.0288		
**Normality Test (Shapiro–Wilk)**	**Statistic (W)**	***p***		
	0.928	0.014		
**Independent Variables**	**Estimate**	**SE**	**t**	***p***
**Intercept ᵃ**	139.061	5.3596	25.7593	<0 .001
**Age**	0.00954	0.0967	0.0986	0.922
**Gender:**				
**F—M**	0.09885	2.3094	0.0428	0.966
**Length Since Initial COVID-19 Infection**	−0.13636	0.1387	−0.9832	0.332

Correlation between the independent and the dependent variable, *R*; The proportion of variance in the dependent variable that can be explained by the independent variable, *R*^2^; Normality of the dependent variable, *Shapiro–Wilk Statistic (W) and p value*; Reference Level, *Intercept ᵃ*; Standard Error, *SE*; The number of standard errors the estimated coefficient is away from the hypothesised value, *t;* Determines if there is a significant relationship between an independent and dependent variable in the model, *p*.

**Figure A1:**
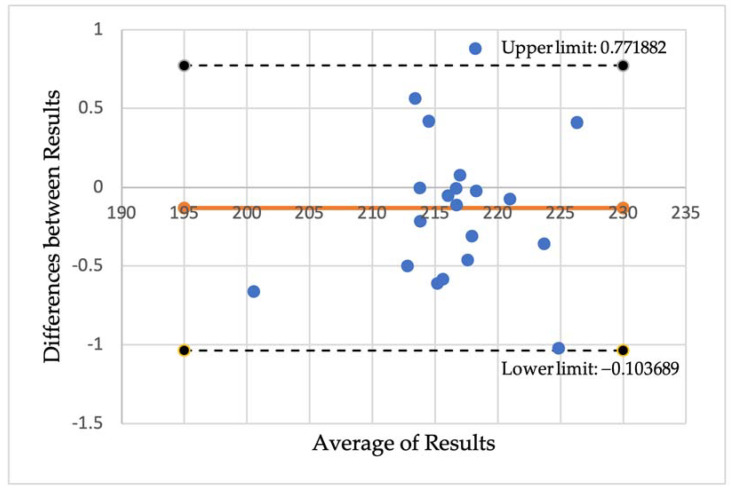
A Bland-Altmann plot demonstrating the difference between the results measuring large vessel intensities using the OCTAVIA software on two occasions by the same assessor.

**Figure A2:**
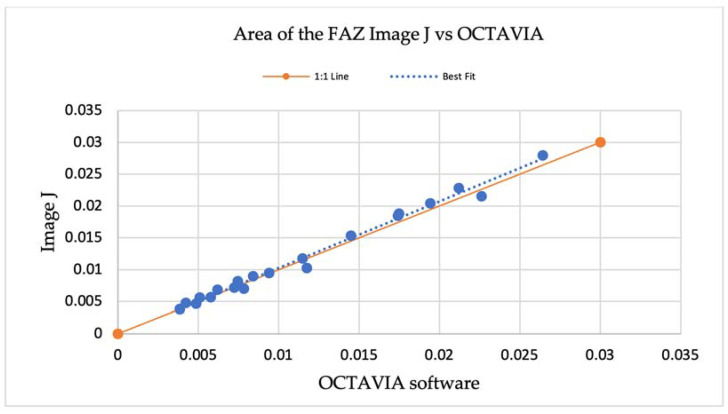
A scatterplot demonstrating the measurements of the area of the foveal avascular zone (FAZ) using Image J and the OCTAVIA software, outlining the relationship between a line of best fit compared to the 1:1 line.

**Figure A3:**
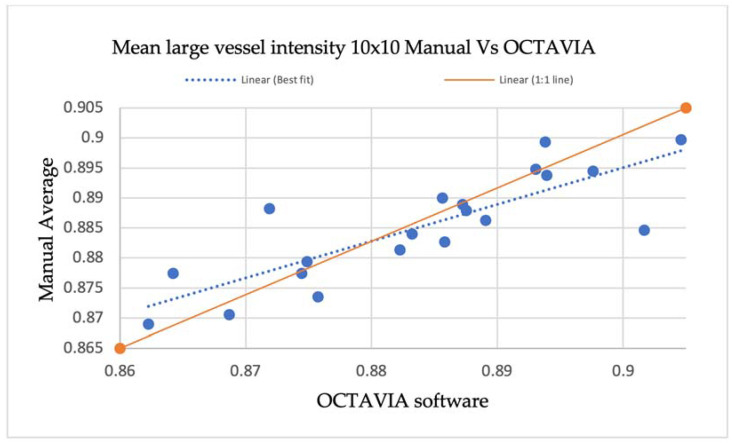
A scatterplot demonstrating manual measurements of large vessel intensities in 10 × 10 OCT-A images compared with OCTAVIA software, outlining the relationship between the line of best fit and 1:1 line.

## Data Availability

All data generated or analysed during this study are included in this published article. The datasets generated during and/or analysed during the current study are available from the corresponding author on reasonable request.

## Appendix A

### Appendix A.1. Reliability and Validity of the Software

To verify the utility of the OCTAVIA software, we appraised the reliability by assessing its automated large vessel intensity measurement in the 10 × 10 mm images of 20 participants on two separate occasions by the same examiner (T.A., a medical retina consultant). This included the manual pre-processing steps involving removal of artefacts. Data is shown below in Table A1.

A Pearson correlation coefficient between the two sets of measurements of 0.9963 was found, demonstrating excellent reliability. A Bland-Altman plot to graphically examine the repeatability was also plotted, shown in Figure A1, which also indicated high reliability. Using jamovi, additional reliability statistics were obtained which were also found to be supportive of our results (Cronbach’s α score of 0.998 and McDonalds’s ω score of 0.997).

**Table A1:** Measurement of large vessel intensities in the 10 × 10 mm OCT-A images of 20 participants, calculated twice to evaluate reliability.

	OCTAVIA1	OCTAVIA2	Mean	Difference
1	200.2152	200.8782	200.546715	−0.66295
2	214.7086	214.2896	214.4990603	0.418981
3	214.8738	215.4842	215.1789925	−0.61038
4	226.5071	226.0975	226.3023362	0.409616
5	224.3287	225.3507	224.8396983	−1.02203
6	213.7813	213.7845	213.7829488	−0.0032
7	223.4939	223.8539	223.6739096	−0.36005
8	216.0177	216.0692	216.0434456	−0.05153
9	215.3221	215.9063	215.6141776	−0.58424
10	217.7659	218.0761	217.9210217	−0.31021
11	212.5384	213.0391	212.7887705	−0.50068
12	216.6652	216.777	216.7211303	−0.11176
13	216.6811	216.6882	216.6846339	−0.00713
14	213.7026	213.9167	213.8096195	−0.2141
15	218.6594	217.7792	218.2192833	0.880265
16	220.9376	221.0125	220.9750348	−0.07491
17	217.0157	216.9396	216.9776428	0.076176
18	217.3771	217.8395	217.608298	−0.46247
19	213.694	213.1311	213.4125719	0.562955
20	218.2591	218.2816	218.2703393	−0.02244

To measure the validity of the OCTAVIA software, Image J was utilised to manually measure the area of the FAZ, comparing this with the measure outputted from the automated OCTAVIA software in the same 20 participants. Data is shown below in Table A2. This was demonstrated in a scatterplot, Figure A2, illustrating that the line of best fit for the relationship between the manual and automatic measurements was not significantly different from the 1:1 line (where a perfect match of both measurements would lie).

**Table A2:** Measurement of the area of the foveal avascular zone (FAZ) using the OCTAVIA software vs manual assessment utilising image J.

	OCTAVIA	Image J
1	0.005788	0.005673325
2	0.0212	0.0227944
3	0.014488	0.01531618
4	0.026428	0.027984508
5	0.017475	0.01882717
6	0.006208	0.00685339
7	0.019427	0.02044105
8	0.007463	0.00819081
9	0.008405	0.00898651
10	0.017428	0.0184428
11	0.007847	0.0070062
12	0.007238	0.007172576
13	0.022608	0.02153121
14	0.004226	0.004765234
15	0.00386	0.00383916
16	0.009413	0.009449548
17	0.004861	0.004664085
18	0.011756	0.010274797
19	0.005088	0.005610388
20	0.011491	0.011762485

An additional evaluation of validity was performed by comparing the measurement of mean large vessel intensities in 10 × 10 mm OCT-A images outputted by the OCTAVIA software with manual measurements made using large vessel data points in MATLAB^®®^ 2021. In the 10 × 10 mm OCT-A images of 20 study participants, three large vessels were selected, and data corresponding with vessel intensity was obtained by selecting two points at the centre of the vessel and two near the margin in each vessel. The data was noted in a Microsoft^®^ Excel^®^ spreadsheet and a total average was calculated to compare against OCTAVIA measurements, shown in Table A3. The results are also demonstrated in a scatter plot, Figure A3, illustrating no significant differences between the line of best fit and 1:1 line.

**Table A3:** Data points corresponding with vessel intensities measured in three large vessels in the 10 × 10 mm OCT-A images of 20 participants using MATLAB, compared with measurements from the OCTAVIA algorithm.

	Large Vessel 1				Large Vessel 2				Large Vessel 3				Manual Average	OCTAVIA
	Centre	Centre	Margin	Margin	Centre	Centre	Margin	Margin	Centre	Centre	Margin	Margin		
1	1	0.929412	0.709804	0.788235	0.92549	1	0.815686	0.792157	0.941176	1	0.85098	0.839216	0.882679667	0.885820538
2	0.913725	1	0.878431	0.768627	0.933333	0.835294	0.898039	0.890196	0.92549	1	0.85098	0.843137	0.894771	0.893043548
3	0.894118	0.945098	0.745098	0.996078	0.933333	0.733333	0.803922	0.913725	0.901961	1	0.784314	0.964706	0.8846405	0.90166909
4	1	0.929412	0.72549	0.819608	0.866667	0.945098	0.8	0.941176	0.901961	1	0.854902	0.85098	0.8862745	0.889066606
5	1	0.933333	0.882353	0.831373	1	0.988235	0.823529	0.843137	0.878431	0.976471	0.803922	0.831372	0.899346333	0.893814466
6	1	0.964706	0.87451	0.752941	0.909804	0.917647	0.870588	0.843137	0.968627	0.929412	0.811765	0.764706	0.883986917	0.883226069
7	0.984314	0.835294	0.819608	0.882353	1	0.988235	0.847059	0.784314	1	0.972549	0.768627	0.784314	0.888888917	0.887244315
8	0.980392	1	0.796078	0.839216	1	0.905882	0.870588	0.709804	0.976471	0.811765	0.85098	0.741176	0.873529333	0.875732357
9	0.92549	1	0.764706	0.839216	1	0.886275	0.7333333	0.827451	0.972549	1	0.827451	0.752941	0.877451025	0.864223212
10	0.933333	0.898039	0.831373	0.879588	0.992157	0.964706	0.858824	0.803922	0.976471	0.972549	0.815686	0.752941	0.88996575	0.885640208
11	0.984314	0.960784	0.815686	0.776471	0.85098	1	0.831373	0.85098	0.905882	0.988235	0.713725	0.768627	0.870588083	0.868688789
12	1	0.945098	0.823529	0.827451	0.996078	0.992157	0.745098	0.784314	0.921569	0.992157	0.784314	0.847059	0.888235333	0.871880492
13	0.917647	0.968627	0.894118	0.780392	1	0.905882	0.898039	0.760784	0.811765	1	0.866667	0.72549	0.877450917	0.874451774
14	0.905882	0.886275	0.878431	0.870588	1	1	0.8	0.780392	0.905882	1	0.807843	0.717647	0.879411667	0.874872568
15	1	0.976471	0.878431	0.835294	0.976471	0.882353	0.760784	0.87451	0.968627	0.968627	0.796078	0.807843	0.89379075	0.893920821
16	0.87451	0.937255	0.835294	0.709804	0.937255	0.929412	0.847059	0.862745	1	0.972549	0.886275	0.784314	0.881372667	0.882249605
17	0.980392	1	0.878431	0.839216	1	0.94902	0.827451	0.752941	0.815686	0.894118	0.894118	0.823529	0.8879085	0.8875187
18	1	0.913725	0.85098	0.843137	0.968627	0.776471	0.87451	0.862745	0.956863	0.917647	0.737255	0.72549	0.868954167	0.862268732
19	1	1	0.815686	0.94902	0.996078	0.92549	0.764706	0.764706	1	0.866667	0.796078	0.917647	0.899673167	0.90460477
20	0.996078	1	0.890196	0.792157	0.882353	1	0.835294	0.745098	0.992157	1	0.768627	0.831373	0.894444417	0.897619232

### Appendix A.2. Sample Size Calculation

The sample size for this study was calculated based on data collected in a previous study on diabetic retinopathy and OCT-Angiography [79]. The method was as follows:

Typically, we set:

$$\begin{array}{c} \alpha \;=\;0.05\;\text{so}\;Z_{\alpha /2}\;=\;1.960\\ \beta \;=\;0.20\;\text{so}\;Z_{\beta /2}\;=\;0.8416\\ \text{which}\;\text{gives}:\\ n_{g}\;≐\;2{\left(z_{\alpha /2}+z_{\beta }\right)}^{2}\;{\left(\frac{\sigma }{\mu_{1}-\mu_{2}}\right)}^{2}\\ ≐\;2\;\times \;{\left(1.960+0.8416\right)}^{2}\;{\left(\frac{\sigma }{\mu_{1}-\mu_{2}}\right)}^{2}\\ ≐\;2\;\times \;7.849\;{\left(\frac{\sigma }{\mu_{1}-\mu_{2}}\right)}^{2} \end{array}$$

σ is the population standard deviation. µ_1_−µ_2_ is the smallest difference with scientific significance. The sample size for this study is based on the sample size for the key metric, mean capillary intensity. The mean capillary intensity of a normal patient from the study was 98.69, shown in Table A4, with σ = 6.23. The difference between mean capillary intensities in Normal vs Diabetic patients with no retinopathy suggested a clinically important difference in this metric, µ_1_−µ_2_ = 98.69−94.22 = 4.47. Therefore: $2\times 7.849\;{\left(\frac{6.23}{4.47}\right)}^{2}=30.49$ ≈ 31. A minimum of 31 in each group was needed, hence, we aimed to have 40 participants in each of our study cohorts (PCS and controls).

**Table A4:** Table excerpt from Aslam et al.’s study of optical coherence tomography angiography in diabetic retinopathy [79] demonstrating the strategy utilised in the calculation of sample size for this study.

Variable	Statistic	Normal, N (*n* = 49)	Diabetic No Retinopathy, DnR (*n* = 50)	Diabetic with Retinopathy, DR (*n* = 53)
Sex		Male 32, Female 17	Male 36, Female 14	Male 43, Female 10
Age	Mean (SD)	57.14 (13.56)	61.06 (12.77)	58.38 (13.06)
	Median	57.0	61.5	60
Best Corrected Visual Acuity (BCVA)	Mean (SD)	85 (19.66)	96.36 (7.39)	86.17 (13.3
	Median	95	95	90
Mean Vessel Intensity	Mean (SD)	180.65 (6.43)	181.38 (6.04)	179.28 (7.45)
	Median	182.0	183.5	181.0
Mean Capillary Intensity	Mean (SD)	98.69 (6.23)	94.22 (5.41)	93.47 (6.03)
	Median	99	95	94

Standard Deviation, *SD*.

## Appendix B

A summary of studies examining OCT-A and SD-OCT images in patients following COVID-19 Infection can be found in Table A5 and Table A6.

**Table A5:** A summary of studies pertaining to OCT-Angiography and SD-OCT macula findings in patients following COVID-19 infection.

Study	Time Following Initial SARS-CoV-2 Infection	Severity of Infection	Size (Case, Control)	Key Findings (Statistically Significant)
**OCT-Angiography: Studies Demonstrating Reduction in Vessel Densities in Patients with a History of COVID-19 Infection**				
Zapata et al., 2022 [62]	≤90 days (3 months)	Mild, Moderate, Severe	24, 24, 21, 27	Reduced VDs in the SCP of patients with moderate and severe disease, compared to mild disease and control subjects.
Turker et al., 2021 [63]	7 days following hospital discharge	Moderate	27, 27	Reduced VD in SCP and DCP regions. No difference in area of the FAZ.
Abrishami et al., 2021 [64]	≥14 days following recovery	Moderate, Severe	31, 23	Reduced VD in the SCP and DCP. No difference in area of the FAZ.
Gonzalez-Zamora et al., 2021 [65]	14 days following hospital discharge	Severe	25, 25	Reduced VD in SCP and DCP regions. Enlargement of FAZ area.
Hazar et al., 2021 [66]	≈30 days (1 month) following hospital discharge	Mild, Moderate	50, 55	Reduced VD in SCP and DCP regions. No difference in area of the FAZ.
Guemes Villahoz et al., 2021 [67]	88 days following initial diagnosis	Moderate, Severe	66 (19, 47), 29	Reduction in VDs in the SCP and reduced perfusion density in the fovea. No difference in the area of the FAZ.
Rodman et al., 2021 [68]	-	Mild	18, 18	Reduced VDs in regions of the SCP.
Yilmaz Cebi et al., 2022 [69]	67–86 days	Mild, Moderate	52, 42	Reduced VDs in SCP and DCP.
Cetinkaya et al., 2022 [70]	≈30 days following hospital discharge	Moderate	45, 45	Reduced VDs in SCP, DCP, and CC.
Abrishami et al., 2022 [71]	7 days, 1 month, 3 months	Moderate, Severe	18 (follow-up study)	Reduced VDs in the SCP and DCP, no difference in the area of the FAZ.
Nageeb Louz et al., 2022 [72]	30–120 days (1–4 months)	Mild, Moderate, Severe	45, 45	Reduced VDs in the SCP, DCP, and enlargement of FAZ.
Cennamo et al., 2021 [73]	180 days (6 months)	Moderate	40, 40	Reduced VDs in SCP, RPC, DCP. RNFL thickness reduced.
Bilbao-Malave V et al., 2021 [74]	6 months from hospital discharge	Severe	17 (follow-up study)	Reduced VDs in SCP and DCP, enlargement of FAZ, Thinner GCL and RNFL.
Banderas Garcia S et al., 2022 [75]	8 months after initial infection	Mild, Moderate, Severe	75, 19	Reduced VDs in the SCP and DCP of patients with moderate and severe disease, compared to mild disease and control subjects. Enlargement of FAZ in patients with more severe disease.
**OCT-Angiography: Studies demonstrating no difference in vessel densities in patients with a history of COVID-19 infection**				
Savastano et al., 2021 [93]	1 month following hospital discharge	Moderate	70, 22	No differences in VD and VP in the SCP and DCP.
Szkodny et al., 2021 [94]	1–4 months following infection	Mild, Moderate	156, 98	No differences in the VDs of the SVP, area of the FAZ, macular RNFL thickness, and central macular thickness.
Kal M et al., 2022 [95]	8 weeks following hospital discharge	Severe	63, 45	No difference in the VDs in SCP or DCP between the two groups. Reduced VD in the foveal CC. Enlargement of area of FAZ.
Chiosi F et al., 2022 [96]	1 month following recovery from infection	Mild, Moderate, Severe	142, 60	No difference in the VDs in SCP. Reduced VD in the DCP and CC.
**OCT-Angiography: Studies demonstrating increase in vessel densities in patients with a history of COVID-19 infection**				
Naderi Beni A et al., 2022 [58]	40–95 days following initial infection	Moderate	51, 37	Increased VDs in the SCP and DCP. Increased peripapillary RNFL thickness.
**OCT macula structural retinal findings in patients with a history of COVID-19 infection**				
Sim et al., 2021 [52]	16.1 ± 3.6 days	Mild	108, 0	Microhaemorrhages, retinal vascular tortuosity, cotton wool spots, hyper-reflective plaques in the GCL-IPL.
Marinho et al., 2020 [54]	11–33 days	Mild to Moderate	12	Hyper-reflective plaques in the GCL-IPL, cotton wool spots, microhaemorrhages.
Burgos-Blasco et al., 2022 [57]	4 weeks following recovery	Mild, Moderate, Severe	90, 70	Increased peri papillary RNFL and macular GCL thickness (more significant in patients with anosmia and ageusia) and decreased macular RNFL thickness.
Oren et al., 2021 [59]	14–30 days	Mild	35, 25	Increased central macular thickness, decreased GCL and INL thickness.
Mavi et al., 2022 [85]	2–8 weeks	Moderate	63 (30 hospitalised), 59	Increased central foveal, mean outer nuclear layer, mean peri papillary RNFL thickness.
Ugurlu A et al., 2022 [86]	29–45 days	Moderate, Severe	129, 130	No difference between COVID-19 and controls. Thinner macular RNFL and GCL in patients with neurological symptoms within the COVID-19 cohort. Reduced VD in SCP, DCP, RPCP, enlargement of FAZ area, reduction of FAZ circularity in symptomatic COVID-10 patients.
Taskiran-Sag et al., 2022 [87]	113 ± 62 days following recovery from infection	Mild, Moderate	40, 40	No difference between GCL thickness between COVID-19 and controls. Thinner macular GCL in patients with neuro-cognitive symptoms.

Choriocapillaris, *CC*; Cotton wool spots, *CWS*; Deep capillary plexus; *DCP*; Foveal avascular zone, *FAZ*; Ganglion cell layer; *GCL*; Inner nuclear layer, *INL*; Inner plexiform layer, *IPL*; Retinal nerve fibre layer, *RNFL*; Radial peri papillary capillary plexus, *RPCP*; radial peripapillary capillaries, *RPC*; Superficial capillary plexus, *SCP*; Vessel density, *VD*; Vessel perfusion, *VP*.

**Table A6:** A Summary of studies pertaining to OCT-Angiography and SD-OCT macula findings in patients with post-COVID-19 syndrome.

Study	Time Following Initial SARS-CoV-2 Infection	Severity of Infection	Size (Case, Control)	Key Findings (Statistically Significant)
Kanra AY et al., 2022 [88]	4.3 ± 2.7 months (1–12)	Mild to Moderate	20, 23	Thinning in segments of the macular RNFL, GCL, and IPL.
Schlick et al., 2022 [89]	231 ± 111 days (7.59 ± 3.65 months)	-	173, 28	Reduced VDs in ICP, no difference in SVP, DCP. Females with PCS had lower VDs in SVP than males. PCS participants with CF had lower VDs in SVP than those without.

Ganglion cell layer, *GCL*; Inner Plexiform Layer, *IPL*; Intermediate capillary plexus, *ICP*; Post COVID-19 syndrome, *PCS*; Retinal nerve fibre layer, *RNFL*; Vessel density, *VD*.
